# Late Seizures in Postpartum Women After Cesarean Section: Differential Diagnosis Between Postpartum Eclampsia and Post-dural Puncture Eclampsia

**DOI:** 10.7759/cureus.95157

**Published:** 2025-10-22

**Authors:** Jaime Miguel Abreu, Mafalda Neves, Rita Rato, Diana Silva, Nulita Lourenço

**Affiliations:** 1 Polyvalent Intensive Care Unit, Unidade Local de Saúde de Castelo Branco, Castelo Branco, PRT; 2 Department of Medical Sciences, Health Science Faculty, Beira Interior University, Castelo Branco, PRT

**Keywords:** magnesium sulfate, medical intensive care unit, post-dural puncture headache, postpartum eclampsia, puerperium, seizure

## Abstract

Postpartum eclampsia is an uncommon but potentially life-threatening complication that can occur up to six weeks after delivery, even in women without prior hypertension. Post-dural puncture headache (PDPH), a frequent adverse effect of spinal anesthesia, typically presents as a postural headache and, in rare cases, may progress to seizures. The clinical overlap between these two conditions poses a significant diagnostic challenge. We report the case of a 25-year-old woman who underwent a cesarean section under spinal anesthesia and developed a headache immediately postpartum, which worsened on day four with generalized tonic-clonic seizures. Cranial CT and CSF analysis were unremarkable. During her intensive care stay, she experienced a hypertensive episode, prompting initiation of magnesium sulfate, levetiracetam, and antihypertensive therapy. Clinical evolution favored the diagnosis of late postpartum eclampsia, despite initial features suggestive of PDPH. Differentiating postpartum eclampsia from PDPH is complex, as both may present with headache and seizures. Hypertension in eclampsia can be intermittent, and proteinuria is not always present, making early clinical suspicion and magnesium sulfate administration critical. PDPH, usually benign, can rarely lead to seizures due to CSF hypotension. Although rare, the coexistence of both conditions cannot be excluded. This case underscores the importance of vigilant postpartum monitoring and prompt evaluation of severe headaches with neurological symptoms. Early recognition and a multidisciplinary approach are essential to minimize complications and ensure maternal safety.

## Introduction

Neurological complications in the puerperium are responsible for significant maternal morbidity. Postpartum eclampsia can manifest up to six weeks after delivery, with up to 16% of cases occurring in this late period [1,2]. Although most episodes occur within the first 48 hours, studies show that up to 44% of affected women are previously normotensive [1,3]. The pathophysiology involves endothelial dysfunction, cerebral vasospasm, and vasogenic edema, often associated with posterior reversible encephalopathy syndrome (PRES) [4].

Post-dural puncture headache (PDPH), in turn, is a relatively frequent complication of spinal anesthesia, with an estimated incidence between 1% and 10% [5]. It is characterized by a postural headache, accompanied by neck pain, nausea, and photophobia. Although typically self-limited, there are reports of serious complications, including seizures and subdural hematomas [6,7].

When faced with a postpartum woman with headache and seizures, the differential diagnosis between late-onset eclampsia and PDPH must be established quickly, given the distinct and potentially lifesaving therapeutic implications.

## Case presentation

A 25-year-old woman of African ethnicity, G2P0, with no relevant personal history, began pregnancy monitoring at 15 weeks of gestation. All routine exams and supplements during the gestational period were normal, including folic acid and iron.

She underwent a cesarean section for fetopelvic disproportion during labor at 40+1 weeks, under spinal anesthesia, without complications. A male newborn weighing 2635 g was delivered, with an Apgar score of 8/9/10. The initial postpartum course was uneventful, and both the mother and newborn were discharged home on the third day.

On the fourth day, she presented to the obstetric emergency department with a severe headache and neck pain, which were persistent but had worsened that day, associated with prostration.

In a multidisciplinary discussion with anesthesiology and intensive care medicine, a diagnosis of PDPH was initially considered, although this diagnosis is uncommon. Following this discussion, cranial and thoraco-abdomino-pelvic CT scans were performed, both of which showed no abnormalities. During the scan, she experienced two episodes of a generalized tonic-clonic seizure (GTCS) and was administered sequential doses of diazepam (5 mg + 5 mg) and propofol (5 mg + 30 mg). Magnesium sulfate therapy was initiated, with no further seizures observed.

The patient was admitted to the ICU, where a lumbar puncture was performed, yielding crystal clear CSF. Microbiological, cytological, and biochemical analyses were within normal limits. Seizure prophylaxis was started with levetiracetam 1 g every 12 hours.

After admission to the ICU, the patient developed severe hypertension (systolic blood pressure: >200 mmHg), requiring isosorbide dinitrate and labetalol infusions, an oral calcium channel blocker, and a diuretic. Based on the clinical course, a diagnosis of late-onset eclampsia was established.

## Discussion

The evaluation of headache with seizures in the puerperium requires the rapid exclusion of potentially fatal secondary causes.

Late-onset eclampsia can occur up to six weeks postpartum, even in normotensive women [1,2]. Described cases report the onset of seizures up to 16 days after delivery, with the diagnosis supported by findings of PRES on neuroimaging [3,4,8]. The absence of severe hypertension or proteinuria does not exclude the diagnosis [2]. Treatment is based on the administration of magnesium sulfate and strict blood pressure control [2].

PDPH results from the loss of CSF after a dural puncture, leading to intracranial hypotension and a consequent postural headache [5]. Although generally benign, it can be complicated by seizures, subdural hematomas, or reversible cerebral vasoconstriction syndrome [6,7,9]. Initial treatment is conservative, but an epidural blood patch is considered the appropriate treatment in persistent cases [5].

In the present case, the history of spinal anesthesia and the early onset of headache suggested PDPH. However, the progression with recurrent seizures and a hypertensive episode supported the diagnosis of late-onset eclampsia, reinforced by the response to magnesium sulfate. The coexistence of the two conditions cannot be excluded, as there are reports of an interaction between CSF hypotension and convulsive seizures in the puerperium [7,9]. The authors aimed to summarize the key clinical features in a comparative table, facilitating the distinction between PDPH and late-onset eclampsia in the postpartum period (Table 1). Importantly, the hospital did not have access to an MRI to confirm the presence of PRES, which limited the diagnostic workup and highlights the challenge of managing such cases in resource-constrained settings.

**Table 1:** Differential diagnosis: PDPH vs. late-onset eclampsia GTCS, generalized tonic-clonic seizure; PDPH, post-dural puncture headache

Feature	PDPH	Late-onset eclampsia
Onset	Typically within 48-72 hours postpartum [5]	Up to six weeks postpartum [1,3]
Symptoms	Postural headache, neck stiffness, and nausea [5]	Severe headache, seizures, and visual disturbances [4,8]
Neurological signs	Rare; seizures may occur in complicated cases [6,7]	GTCSs and altered mental status [3]
Risk factors	Recent spinal or epidural anesthesia [5]	May occur in normotensive women [1,2]
Blood pressure	Typically normal	Often elevated; may be sudden and severe [1]
CSF findings	May show low pressure if measured [5]	Usually normal [4]
Imaging findings	Often normal; may show signs of intracranial hypotension [7]	PRES may be present on MRI [8]
Treatment	Conservative (hydration and caffeine); epidural blood patch if persistent [5]	Magnesium sulfate, antihypertensives, and ICU monitoring [1,2]

This case is particularly unique because the patient initially lacked the classic features of eclampsia, such as sustained severe hypertension or proteinuria, making the diagnosis less straightforward and emphasizing the need for clinical vigilance.

Therefore, intense headaches and seizures in postpartum women should not be attributed exclusively to a dural puncture. The suspicion of late-onset eclampsia must be maintained, regardless of the initial presence of hypertension or proteinuria. In similar scenarios, we recommend that patients presenting with postpartum headache undergo prolonged monitoring and additional evaluation before attributing symptoms solely to PDPH in order to avoid delayed recognition of potentially life-threatening conditions.

## Conclusions

The differential diagnosis between late-onset eclampsia and PDPH is challenging, given the overlap of clinical manifestations. The approach must be immediate, with the early initiation of magnesium sulfate and strict control of blood pressure whenever eclampsia is suspected. This case reinforces the importance of clinical vigilance during the puerperium and the early investigation of headaches with neurological symptoms, ensuring greater maternal safety.
